# Diverse of Erythropoiesis Responding to Hypoxia and Low Environmental Temperature in Vertebrates

**DOI:** 10.1155/2015/747052

**Published:** 2015-10-18

**Authors:** Shun Maekawa, Takashi Kato

**Affiliations:** ^1^Department of Biology, School of Education, Waseda University, 2-2 Wakamatsu, Shinjuku, Tokyo 162-8480, Japan; ^2^Graduate School of Advanced Science and Engineering, Waseda University, 2-2 Wakamatsu, Shinjuku, Tokyo 162-8480, Japan

## Abstract

Erythrocytes are responsible for transporting oxygen to tissue and are essential for the survival of almost all vertebrate animals. Circulating erythrocyte counts are tightly regulated and respond to erythrocyte mass and oxygen tension. Since the discovery of erythropoietin, the erythropoietic responses to environment and tissue oxygen tension have been investigated in mice and human. Moreover, it has recently become increasingly clear that various environmental stresses could induce the erythropoiesis via various modulating systems, while all vertebrates live in various environments and habitually adapt to environmental stress. Therefore, it is considered that investigations of erythropoiesis in vertebrates provide a lead to the various erythropoietic responses to environmental stress. This paper comparatively introduces the present understanding of erythropoiesis in vertebrates. Indeed, there is a wide range of variations in vertebrates' erythropoiesis. This paper also focused on erythropoietic responses to environmental stress, hypoxia, and lowered temperature in vertebrates.

## 1. Introduction

Erythrocytes are responsible for transporting oxygen to tissue and are essential for the survival of almost all vertebrate animals. All vertebrates adapt to various environments. Therefore, investigations of erythropoiesis in vertebrates are considered vital to address the various erythropoietic responses to environmental stress. The vertebrates that lack hemoglobin and erythrocytes are the larvae of eels and a few Antarctic fish of the family Channichthyidae [1]. Moreover, only mammalian species in vertebrates have anucleate erythrocytes, while erythrocytes of nonmammalian species have nuclei and a shape of an ellipse. The advantage of anucleate erythrocyte is explained by flexibility in developed capillary and incensement of surface area for oxygen binding. Moreover, it has been demonstrated that the erythropoietic systems are diverse among vertebrates (Table 1). Although the science behind them is less than clear-cut, such a wide-ranging difference should bring diversity to the hematopoietic systems for responding to environmental stress. To address the description of erythropoietic functions, this paper introduces the present understanding of erythropoiesis in vertebrates, focusing on erythropoietic responses to environmental stress, hypoxia, and lowered temperature.

## 2. Diversity of Erythropoiesis

### 2.1. Erythropoietin

In 1977, Miyake and his colleges isolated native human erythropoietin (EPO) from urine of patients with aplastic anemia [2]. In mammalian species, EPO expression and secretion in the kidney are induced by hypoxia, and then highly glycosylated EPO circulates to the bone marrow in adult mammals to stimulate the proliferation and differentiation of EPO receptor expressing erythroid progenitors in an endocrine manner [3–5]. Subsequently, this notion is widely understood as a central mechanism of the production of mammalian red blood cells [6, 7]. Long afterward, EPO gene was identified for the first time in nonmammalian species and was reported in pufferfish,* Fugu rubripes* [8], and then zebrafish [9, 10]. The primary sites of EPO expression in fish was unexpectedly found in the heart [8]; however the biological function of the heart EPO has not been directly elucidated. We have reported the identification and biological properties of EPO [11, 12] and EPO receptors (EPOR) [13, 14] in the African clawed frogs,* Xenopus laevis*. The highest expression of EPO in* Xenopus laevis* was found in the lung and lesser in the liver [12]. It was our surprise that* Xenopus* EPO, unlike in human and murine EPO, lacks N-glycosylation that is essential for* in vivo* stability and biological activity in the circulation. When* N*-linked carbohydrates were artificially introduced to* Xenopus* EPO, however,* in vitro* activity was not interfered [15]. We also demonstrated that recombinant* Xenopus *EPO induces proliferation of human cell lines expressing* Xenopus* EPOR as well as human EPOR, despite the 38% amino acid identity shared between* Xenopus* EPO and human EPO [12]. In addition, an essential tertiary structure of the ligand-receptor (EPO-EPOR) is reserved and shared among those species [11]. Because of these findings, the ligand specificity should not be determined solely by primary amino acid sequences.

### 2.2. Erythropoietic Organ

Erythropoietic organs in vertebrates are various. In mammals, erythropoiesis mainly occurs in bone marrow at their adult stage. Additionally, the spleen also causes erythropoiesis in some rodents [16–18]. The studies of searching hematopoietic organs in nonmammals have been performed through the ages [19–25]. Although most investigations are approached though cellular morphological observation, there are reports using molecular based technics during recent years.

In teleost fish, hematopoiesis occurs in the kidney. A study of brown trout has indicated that the spleen is also an erythropoietic organ [19]. In zebrafish study, reporter transgenesis takes the advantage to isolate and characterize the hematopoietic progenitor cells. An approach using* gata1* reporter transgene and flow cytometry to separate the erythroblastic cells from the kidney has been developed in the* gata1* reporter transgenic models [26–28]. Kidney of zebrafish comprehends the erythroid progenitor cells that induced proliferation and differentiation by recombinant EPO* in vitro *[29].

In aquatic amphibian,* Xenopus laevis*, we recently demonstrated that erythropoiesis mainly occurs in the adult liver to reveal the presence of CFU-E [14, 30]. The EPOR positive cells are found predominantly on the inner wall of hepatic sinusoids and increased during erythropoietic stress [14]. In northern crested newt, erythroblastic cells are observed in the heart aside from the spleen after phenylhydrazine- (PHZ-) induced anemia. Although we also observed erythroblasts in periphery during erythropoietic stress induced by PHZ, erythroblasts could not be observed in periphery in normal state [13]. Therefore, it is suggested that these erythroblast cells that are observed in periphery are released from erythropoietic organ responding to erythropoietic stress.

While, in bullfrog (*Lithobates catesbeianus*), terrestrial amphibian, de Abreu Manso and his colleagues reveal that hematopoietic cells expressing CD34, CD117, and EPOR exist in the adult bone marrow of vertebrae, femur, and fingers and in the kidney detected by immunohistochemistry [31], in reptiles and avian, bone marrow is the primary erythropoietic site [21, 22, 24, 32, 33]. In chicken, erythroid progenitor assay is developed well using bone marrow cells [33]. Taken together, in aquatic amphibian, erythropoiesis mainly occurs in kidney, spleen and/or liver. From terrestrial amphibian, erythropoiesis is found in bone marrow, while, in aquatic mammalian bottlenose dolphins (*Tursiops truncatus*), bone marrow mononuclear cells contain hematopoietic progenitor cells [34].

### 2.3. Fate of Circulating Erythrocytes

Erythrocyte is damaged little by little during circulation and destructed eventually. In adult human, the life span of erythrocyte is about 120 days [35]. The life span is not correlating with the direction of evolution. The erythrocytes lifespans of mice [36], rabbit [37], and chickens [38] are about 40, 55, and 35 days, respectively, which are shorter than human. In amphibian and fish, the life span tends to be longer compared with mammalian species. The life span of erythrocytes in* Xenopus laevis* is about 220 days [39], longer than mouse and human. In ginbuna crucian carp, the erythrocyte lifespan is about 50 days, and labeling erythrocytes are detected in circulation up to 270 days [40]. Biological implication of difference in the erythrocyte life span is not well understood.

In mammals, damaged or senescent erythrocytes are degraded by phagocytes in the liver and spleen [41]. Investigations of erythrocyte destruction sites in nonmammalian species are limited. Our recent work with* Xenopus laevis* showed that erythrocyte destruction mainly occurred in the liver based on the expression of heme degrading enzymes, hemeoxygenase-1, and biliverdin reductase A expression and the accumulation of iron [39].

## 3. Erythropoiesis Response to Environmental Stress

### 3.1. Hypoxia

The primary EPO producing organ is the kidney in adult mammal, while most of the knowledge of hypoxic EPO expression has been based on human hepatoma cell lines. The molecular mechanisms of hypoxia inducible EPO gene expression involve hypoxia inducible transcription factors (HIFs), which are primary transcriptional factors of the hypoxic response [7, 42] and are negatively regulated by the von Hippel-Lindau (VHL) factor [43, 44]. The VHL acts to ubiquitylate the catalytic *α* subunit of HIF and induce their turnover in normoxia due to recognition of a prolyl hydroxylation motif, which is oxygen dependent hydroxylases to modify *α* subunit of HIF. During hypoxic condition, the loss of hydroxylase activity causees the accumulation and transactivation function of HIF-*α* subunit [45]. The most well characterized HIF regulatory region is a liver-specific hypoxia response element (HRE) that is the binding site of HIF-1, which is located within 0.7 kb 3′ of the polyadenylation signal [45]. Recently the analysis of renal EPO expression has been performed and increasingly obvious [46–48]. There are also reports that EPO is detected in various tissues (brain, heart, lung, and testis) and exhibited nonerythropoietic activity [49–51]. Taken together, we have a question if each EPO producing organ could response to hypoxia and promote erythropoiesis or not. To resolve this issue, conditional knockout experiments in which a target gene can be specifically inactivated in specific tissue(s) were performed. The deletion of VHL gene prevents the ubiquitination of hydroxylated HIF-*α* proteins and causes a hypoxic phenotype due to accumulation of HIF-*α* proteins. To demonstrate the contribution of each EPO producing organ, various mice in which VHL can be inactivated tissue specifically were generated using conditional gene targeting technology based on Cre-loxP mediated recombination [52]. Hepatocytes specific deletion of VHL using albumin promoter enhanced hepatic EPO expression and induced polycythemia [53]. Mice with osteoblasts specific knockout of VHL showed the enhancement of EPO expression in bone marrow and erythropoiesis [54]. The expression level of renal EPO was decreased in the mice. Interestingly, it has been reported that renal EPO expression is induced by the variation of oxygen partial pressure in other tissues. Rats with high cerebral pressure exhibit a significant increase in plasma EPO levels [55]. This report suggested that EPO expression in the kidney is induced by a brain stem-derived humoral factor at high intracranial pressure. Boutin et al. showed that HIF-1*α* gene deletion in mice epidermis inhibits renal EPO synthesis in response to hypoxia and epidermal sensing of oxygen is important for the renal EPO synthesis [56]. Based on these observations, it is suggested that each organ have an oxygen sensor independently in mammalian species.

In nonmammalian species, the knowledge of EPO gene expressions responding to hypoxia is limited. In zebrafish, cardiac EPO mRNA expression is moderately upregulated 6.5 h after being exposed at hypoxic environment [9]. HRE in teleost EPO gene locus of fugu has been reported [57]. The fugu HRE is located in the 5′ flanking region of EPO gene locus. However, the HRE is on the opposite strand of DNA, unlike the HRE of EPO gene locus in the human, and it is unclear whether the HRE contributes to response to hypoxia and induces the EPO gene expression or not. We recently have showed that the expression of EPO gene in* Xenopus laevis* is not induced by anemia investigated [12]. The hypoxic response and diversity organs of EPO gene expression in nonmammalian species are subject of future investigation.

Before indicating the existence of EPO, Misher (1893) suggested that hypoxic stress directly stimulates bone marrow and promotes erythropoiesis. Although this hypothesis was rejected by the identification of EPO once, the response of erythroid cells to hypoxia has been recently investigated.* Ex vivo* erythrocytes production from peripheral and cord blood CD34+ cells was enhanced by low oxygen concentration (1.5–5%) exposure [58]. Under hypoxic conditions, the mRNA and protein amounts of delta-aminolevulinate synthase 2 (ALAS2) and GATA-1 (erythroid specific transcriptional factor) were elevated in human erythroleukemic cell line, K562 cells, and erythroid induction cultures of CD34^+^ hematopoietic stem/progenitor cells [59, 60].

It has been reported that the type of hemoglobin is changed by hypoxia stimuli. In human, embryonic, fetal, and adult hemoglobin is sequentially expressed in erythroblasts during developing developments. The binding affinity of hemoglobin is different among the type of hemoglobin. Increasing the oxygen-binding affinity of fetal hemoglobin is relative to that of adult. In erythroid differentiated culture from human hematopoietic progenitor cells, the number of cells with fetal hemoglobin increased, exposed to low O_2_ [61]. A moderate increase of circulating erythrocytes with fetal hemoglobin was observed in human during and after the hypoxia exposure [62]. Erythrocytes with fetal hemoglobin have an advantage to bind more oxygen under hypoxic condition. In amphibian species, hemoglobin switch from the larval to the adult type of hemoglobin has been showed during metamorphosis (from aquatic to terrestrial life) [63, 64]. Because the oxygen dissociation curve in tadpole shifts to left compared with that in adult [65], the tadpole erythrocyte more readily takes up oxygen, so that it is an advantage for tadpoles to live in water with lower oxygen. These reports indicated that hemoglobin switching is one of the important responses to adapt hypoxic environment.

Recently, a type of noncording RNA, micro-RNA (miRNA), has attracted attention for the factor responding to environmental stress. miRNAs are single-stranded RNA molecules approximately 22 nucleotides in length. Each miRNA recognizes target mRNAs via base-pairing interactions and induces the gene silencing. We compared the miRNA expression patterns in UT-7/EPO, UT-7/GM, and UT-7/TPO cell lines which are all differentiated from UT-7 cells by cytokine stimulation and exhibit the same genotype with different phenotypes, and demonstrated some specific miRNAs expressed in erythroid cells [66]. One of them, miR-210, is expressed in the late stage erythroid cells in mouse [66]. miR-210 is highly expressed by hypoxic condition and regulates the iron homeostasis via targeting the iron-sulfur cluster scaffold protein and transferrin receptor [67]. Recently, Sarakul et al. demonstrated that miR-210 induced erythroid differentiation in K562 cells and CD34^+^ hematopoietic stem/progenitor cells [68]. Although seen mostly* in vitro*, these results suggested that erythroid cells could be regulated by low O_2_ condition beyond the EPO response.

### 3.2. Low Temperature

In birds and mammals, endothermic animals process the mechanisms to maintain the body temperature, independent of the environmental temperature. Upon the initial exposure to low temperature, endothermic animals exhibit peripheral vasoconstriction to reduce heat loss from body. Active thermogenesis occurs by means of periodic shivering if heat dissipation exceeds metabolic heat generation [69]. During prolonged exposure to low temperature, nonshivering thermogenesis is enhanced by synthesis and catalysis of ATP in the brown adipose tissue and muscle. Additional metabolic heat generation is accompanied by increased oxygen consumption [69]. Therefore, it is readily hypothesized that erythropoiesis would respond and contribute to adaptation to low environmental temperature.

There are many reports that showed the response of hematopoiesis to environmental temperature. Nine-banded armadillo (*Dasypus novemcinctus*) possesses the active hematopoiesis in the dermal bone marrow of the armor. In winter season, the dermal bone marrow became fatty and showed less hematopoiesis, compared with summer season [70]. The marrow of tailbone in new borne rats has active hematopoiesis and replaced the adipose with maturity. Tavassoli et al. showed that the capability of hematopoiesis was maintained in the tail vertebrae of newborn rats by transposing the tail into the warmer environment of the abdomen [71]. It has been demonstrated that the variation of blood cell counts is induced by low environmental temperature and season change in various vertebrates (Table 2). In rats and chickens acclimated to low temperature, an increase in the number erythrocytes was observed [72, 73]. We have investigated whether increase in circulating erythrocytes induced by low temperature exposure is caused by enhanced erythropoiesis in mice [74]. Mice were exposed to a 5°C environment for 56 days. The blood hematocrit levels (HCT) gradually increased by day 14 and remained high at day 56. The proportion of proerythroblasts in the spleen and bone marrow increased greatly by day 5 of exposure to low temperature. EPO mRNA levels increased in the kidneys, and hypoxia inducible genes were enhanced in the kidney after being exposed to low-temperature environment. These results indicated that erythropoiesis was enhanced, explaining the high level of EPO mRNA expression in the kidney. In addition, the level of oxygen tension in the kidney was decreased after low-temperature exposure. Elevated erythrocyte counts would increase oxygen supply to peripheral tissues for heat production after low-temperature exposure.

In the studies of nine-banded armadillo and newborn rats as described previously, it is considered that hematopoietic effects are caused by hematopoietic organ itself exposed to low temperature. Moreover, cases of anemia or pancytopenia in hypothermic patients have been reported [75–79]. Establishing the hypothermic model in small mammals has been hampered by practical difficulties. We used the African clawed frog;* Xenopus laevis* was used to investigate the cause of hypothermia-induced anemia [39]. Frogs were exposed to low temperature (5°C) for five days and then were put back to 22°C immediately afterwards. One day after exposure to 5°C, erythrocyte count decreased by approximately 30% and then remained at this level for 5 days. Two days after the return to 22°C, erythrocyte count had recovered to initial levels. The rate of destruction of erythrocytes in adult* Xenopus laevis* was estimated to be about 0.45% per day under normal conditions from 220-day erythrocyte lifespan. It was hypothesized that enhancement of erythrocyte degradation caused erythrocytopenia after low-temperature exposure. Primary organ of erythrocyte degradation is the liver in adult* Xenopus laevis*. In the liver, heme oxygenase (HOMX1) and biliverdin reductase (BLVRA) mRNA increased after exposure to low temperature, and then accumulation of iron as a result of heme degradation was observed in the liver. These results indicated that hypothermic anemia was initiated by enhanced peripheral erythrocyte destruction. It is hypothesized that the cause of anemia under hypothermic conditions is a downregulation of erythropoiesis. However, in contrast, we found that EPO mRNA expression was elevated in lung and liver after low-temperature exposure and hepatic erythropoiesis was upregulated. Despite upregulation of erythropoiesis, newly produced erythrocytes are not released to the circulation but appear to remain in the hepatic sinusoid, which could explain the prolonged anemia observed during low-temperature exposure. To investigate the modulated molecules responding to low temperature, moreover, we attempted the proteomics to profile the hepatic proteome in* Xenopus laevis* after exposure to low temperature [80]. Our proteome data suggested that glycolytic and antioxalate systems acted after the accumulation of hepatic iron caused by low-temperature exposure.

The signal passway from sensitive environmental low temperature to erythropoietic change is unclear. However, we demonstrated that the rule of erythropoiesis responding to low environmental temperature is different between endothermic and ectothermic animals through our comparative study.

## 4. Conclusion and Perspective

Since the discovery of EPO, it has been investigated that the expression and secretion of renal EPO which respond to environment and tissue oxygen tension could regulate the circulating erythrocyte counts. Through the study of various vertebrates, it has become increasingly clear that vertebrates possess the unique erythropoietic responses to individual habitat and environmental stress. Recently, the many experimental technologies, such as gene editing and omics, have been developed and are available to not only laboratory animals but also nonlaboratory animals. Therefore, comparative study holds the more potential for enhancing our knowledge of erythropoietic systems. Furthermore, to understand the physiology of the whole organism and/or cell, analyzing cyclopedic modulating molecules induced by environmental stress and understanding the network relationship among these molecules were attempted. Through this analysis, the various systems to link to erythropoiesis will become known.

## Figures and Tables

**Table 1:** Diversity of erythropoiesis in nonmammal.

Species	EPO	Erythropoietic organ		Life span	
	Producing organ	Site	Cell identification	Days	Cell labeling
Fish					
Pufferfish (*Takifugu rubripes*)	Heart [8]				
Zebrafish (*Danio rerio*)	Heart [9, 10]	Kidney	*gata1* reporter transgenesis [26, 27], progenitor assay [29]		
Brown trout (*Salmo trutta*)		Spleen, kidney	Cell morphology [19]		
European perch (*Perca fluviatilis*)		Spleen	Cell morphology [19]		
Common Roach (*Rutilus rutilus*)		Kidney	Cell morphology [19]		
Common carp (*Cyprinus carpio L*)		Kidney	Cell morphology [25]		
Ginbuna crucian carp (*Carassius auratus langsdorfii*)		Kidney (stem cell)	Kidney marrow cell transplantation [81]	270	PKH 26-GL [40]

Amphibian					
African clawed frog (*Xenopus laevis*)	Lung, liver [12]	Liver	Progenitor assay [30], immunohistochemistry [14]	220	Biotin *in vivo* [39]
Bullfrog (*Lithobates catesbeianus*)		Kidney, bone marrow	Immunohistochemistry [31]		
Leopard frog (*Lithobates pipiens*)				200	DFP32 [82]
Great crested newts (*Triturus cristatus*)		Spleen, heart	Cell morphology [20], PHZ-induced anemia [23]		
Ezo salamander (*Hynobius retardatus*)		Spleen	*In situ* hybridization [83]		

Reptiles					
Painted turtle (*Chrysemys picta*)		Bone marrow, kidney, spleen	Fe^59^ incorporation [32]		
European pond turtle (*Emys orbicularis*)		Bone marrow	Cell morphology [21]		
Spanish lizard (*Lacerta hispanica*)		Bone marrow	Cell morphology [22]		
Boa constrictor (*Boa constrictor*)		Bone marrow	Cell morphology [24]		
Corn snake (*Elaphe guttata*)		Bone marrow	Cell morphology [24]		
Brown house snake (*Lamprophis fulaginosus*)		Bone marrow	Cell morphology [24]		
Bothrops jararaca (*Bothrops jararaca*)		Bone marrow, spleen	Cell morphology [24]		

Avian					
Chicken (*Gallus gallus*)		Bone marrow	Progenitor assay [33]	35	Na_2_Cr^51^O_4_ [38]
White Carneaux pigeons (*Columba livia*)				35–45	Na_2_Cr^51^O_4_ [38]
White Pekin duck (*Anas platyrhynchos*)				42	Na_2_Cr^51^O_4_ [38]

**Table 2:** Hematopoietic responses to low environmental temperature and seasons in vertebrates.

Stress	Species	Experimental condition	Peripheral blood cell counts		Hematopoietic observation	Reference
			Increase	Decrease		
Seasonal change	Nine-banded armadillo (*Dasypus novemcinctus*)	Winter			Decrease hematopoietic cells in armor dermal bone marrow	[70]
	Edible frog (*Rana esculenta*)	Winter		RBC, HGB		[84]
	Wisent European bison (*Bison bonasus*)	Summer	RBC size			[85]
	Grass snake (*Natrix natrix*)	Winter		RBC, HGB, HCT		[86]
	Bottlenose dolphin (*Tursiops truncatus*)	Winter		RBC, HGB, HCT		[87]

Low temperature	Leopard frog (*Rana pipiens*)	5°C		HCT	RBC lifespan extends	[88]
	Painted turtle (*Chrysemys picta*)	2°C		HCT		[89]
	Cunningham's skink (*Egernia cunninghami*)	8°C		HCT, HGB		[90]
	Sidewinder (*Crotalus cerastes*)	20°C	RBC, HGB, HCT			[91]
	Fossil catfish (*Heteropneustes fossilis*)	18°C		RBC, HGB, HCT, WBC		[92]
	American bullfrog (*Rana catesbeiana*)	5°C		RBC, WBC, TBC		[93]
	Rat (*Rattus norvegicus*)	5°C	RBC, HGB, HCT			[72]
	Chicken (*Gallus domesticus*)	10°C	HGB, HCT			[73]
	Zebrafish (*Danio rerio*)	17°C			Downregulate erythropoietic genes expression level	[94]
	African clawed frog (*Xenopus laevis*)	5°C		RBC, WBC, TBC	Enhanced hepatic erythrocyte destruction Newly produced erythrocytes are confined to the liver	[39]
	Mouse (*Mus musculus*)	5°C	HCT, HGB		Upregulate erythropoiesis	[74]

Other	European hamster (*Cricetus cricetus*)	Hibernation		WBC, TBC		[95]
	Syrian hamster (*Mesocricetus auratus*)	Hibernation		TBC		[72]
	Rat (*Rattus norvegicus*)	Transposing the tail into the abdomen			Maintain the hematopoiesis in bone marrow of tail vein	[71]

RBC, erythrocyte; HGB, hemoglobin; HCT, hematocrit; WBC, leukocyte; TBC, thrombocyte.
